# Practical Observations on Midwifery, with Cases in Illustration

**Published:** 1843-04

**Authors:** 


### Art. XX.

*Practical Observations on Midwifery, with Cases in illustration.* By John Ramsbotham, M.D. &c. &c. Second Edition, revised.—*London,* 1842. 8vo, pp. 496.

The work now before us is a second and revised edition of a treatise originally published in two parts in 1821 and 1832, condensed into one volume, partly by a diminution in the size of the type, and partly by the introduction of fewer cases. The very nature of the work precludes the possibility of a lengthened analysis. We must, therefore, limit ourselves to a general notice of the contents of the volume, and some of Dr. Ramsbotham's opinions upon points of practical importance. And first, of the ergot of rye. "For a length of time" Dr. Ramsbotham continued sceptical as to the active powers of the ergot, but he is now ready to acknowledge and duly to appreciate its influence. Yet even now he has his doubts, upon a general principle, whether its introduction ought to be hailed as a boon or reprobated as an evil. He fears, in short, and no doubt with good reason, that this agent is very likely to be employed when interference of any kind is unnecessary. The same apprehensions, however, might fairly be expressed with respect to all medical or surgical means. In bad hands the best of remedies may be mischievous. The introductory observations on the act of childbirth, and the chapter on natural labour, are clear and concise, and well worth the perusal of the midwifery student.

Upon the oft-disputed point of the muscularity of the uterus, Dr. Ramsbotham states, "that however authors may write and teachers may talk about the uterine muscles, no such structure is evident to *my* senses. Even the able disquisition of Sir Charles Bell, (Med. Chir. Trans., vol. iv.) on the muscularity of the uterus, does not convince my mind on that subject." Upon this physiological rather than practical question, Dr. Ramsbotham's arguments are plausible. Upon the whole it appears to us that Velpeau takes a fair and satisfactory view of the subject, and he concludes "that it is during pregnancy that we must study the tissue of the uterus for the purpose of determining its nature. It is then only that it is red, and contractile, and fibrous; that it contains a large proportion of fibrine, and that it presents, in a word, every character of the best developed muscular tissue."* If Dr. Ramsbotham is correct in asserting that "certainly the examination of the uterus in the different classes of brute animals throws no light on the doctrine of muscularity," (p. 5,) Dr. W. Hunter was wrong; for he says, "in the quadruped, the cat particularly and the rabbit, the muscular action, or the peristaltic motion of the uterus, is as evidently seen as that of the intestines, when the animal is opened immediately after death."†

The practical comments on and the causes of adhesion of the placenta are very instructive. We quite agree with Dewees, who published an American edition of Dr. Ramsbotham's work as it first appeared, "that by an obedience to the precepts inculcated in this chapter, even a young practitioner may conduct these truly perilous cases to a happy issue; by a neglect of them, an old one may have his victims." Notwithstanding the care with which every writer, and we hope every teacher, on midwifery discusses and details the management of adhesion of the placenta,

* Traité de l'Art des Accouchemens, 1835, tome i. p. 85.

† Anatomical Description of the Gravid Uterus, 1794, p. 26.

it is quite lamentable to reflect upon the daily malpractice exhibited in such instances.

The observations and cases of "retention and disruption of the placenta," and on "relaxation of the uterus after delivery," even after it has seemed to the hand to have acquired a considerable share of contraction and of diminution in size, are equally deserving of attention. In cases of protracted labour, Dr. Ramsbotham objects to the employment of opiates, which are advised by many to relax the rigidity of the soft parts. "Their effects are at the best uncertain, nor do I suppose that they have any tendency to produce relaxation of parts. In large doses they may procure ease from pain, but they also bring about a cessation of uterine action, the return of which is not under control, nor to be ensured at pleasure." (p. 130.) We have several times known the use of the forceps rendered necessary by the injudicious exhibition of opiates during labour, from the complete uterine torpidity that was produced. Many excellent and instructive cases are related of the protraction of labour from various degrees of pelvic deformity and other causes.

The rules that Dr. Ramsbotham lays down for the management of breast and shoulder presentations do not materially differ from those of other writers. In those cases in which turning has been previously but unsuccessfully attempted, "large doses of opiates have been advised, and sometimes administered, with the intention of diminishing uterine power;" of removing some part of that contraction which resists the admission of the hand; but generally without any apparent advantage.

In uterine hemorrhage of the accidental kind, Dr. Ramsbotham strongly advocates the efficacy of rupturing the membranes, and he answers, in our opinion very satisfactorily, the objections that a few writers have urged against the practice. The circumstances which render this expedient necessary are carefully mentioned. Very prudent cautions, too, are given as to the external use of cold. All are probably aware that this is a powerful means of arresting uterine hemorrhage, but many are not aware, —we speak from personal observation—that its employment requires great care.

"But the external use of cold, whether in the form of iced fluids, or of solid ice enveloped in a bladder, requires, in my opinion, considerable discretion, especially under a state of exhaustion. I have found in most cases its temporary application more beneficial than a regular perseverance in its use for a length of time. I therefore generally recommend that it should be applied for a given time, that it should be withdrawn for an equal time, and that it should be repeated; that a natural warmth may take place in the intervals." (p. 272.)

From the brief and slight manner in which Dr. Ramsbotham mentions the use of the plug in uterine hemorrhage, either accidental or unavoidable, we infer that he has but little confidence in its employment. We think, however, that Burns and many other writers have clearly proved the safety and efficacy of this mode of practice in appropriate cases.

The chapters on puerperal convulsions, plural births, abortion, and rupture of the uterus, contain abundant matter that is well worth the attention of the student and young practitioner. Upon the subject of polypus of the uterus, Dr. Ramsbotham must permit us to observe, that the safety of excision, and its general superiority to the ligature, is now almost universally acknowledged by the most experienced surgeons of this and other countries. We have before commented upon this subject.*

* British and Foreign Medical Review, July, 1837, p. 183.

